# High-Entropy Design of Perovskite Quantum Paraelectrics with Improved Dielectric Properties in GHz and THz Bands

**DOI:** 10.1021/acsami.6c04586

**Published:** 2026-06-22

**Authors:** Wanting Hu, Xuyao Tang, Harry Baxter, Vladimir Koval, Krishnarjun Banerjee, Michael J. Reece, Bin Yang, Haixue Yan

**Affiliations:** † School of Engineering and Materials Science, Queen Mary University of London, Mile End Road, London E1 4NS, U.K.; ‡ Faculty of Science and Engineering, 11965 University of Chester, Chester CH2 4NU, U.K.; § Institute of Materials Research, Slovak Academy of Sciences, Watsonova 47, 040 01 Kosice, Slovakia

**Keywords:** high-entropy ceramics, quantum paraelectric, microwave, terahertz spectroscopy, perovskite oxides

## Abstract

High-entropy engineering offers a promising pathway to enhance dielectric permittivity at elevated frequencies in perovskite oxides by increasing compositional complexity. Here, two titanate-based high-entropy quantum paraelectrics, (Ba_0.2_Sr_0.2_Ca_0.2_La_0.2_Na_0.2_)­TiO_3_ (BSCLN) and (Ba_0.2_Sr_0.2_Ca_0.2_La_0.2_K_0.2_)­TiO_3_ (BSCLK) were developed via entropy-driven phase stabilization, and their dielectric responses were systematically investigated from radiofrequency to terahertz regimes. Both materials exhibit spatially distributed local polar modes arising from entropy-induced configurational local polar disorder (LPD) and entropy-enhanced lattice-coupled local polar fluctuation (LPF). In the radiofrequency range, weak- and strong-field permittivity are nearly identical, with the strong-field permittivity remaining ∼ 250, indicating a dominant ionic polarization contribution and excellent dielectric stability. Negative extrapolated Curie temperatures (<0 K), together with the temperature-dependent permittivity behavior, confirm the quantum paraelectric nature of both compositions. In the microwave regime, the high-entropy quantum paraelectrics show significantly higher dielectric permittivity (∼155–189) and lower dielectric loss (∼0.002) compared to BaTiO_3_ and SrTiO_3_, while their terahertz response is governed by entropy-enhanced local polar fluctuations. These results demonstrate that combining high-entropy design with quantum paraelectric functionality provides an effective strategy for developing high-frequency dielectric materials for next-generation electronic devices.

## Graphical abstract

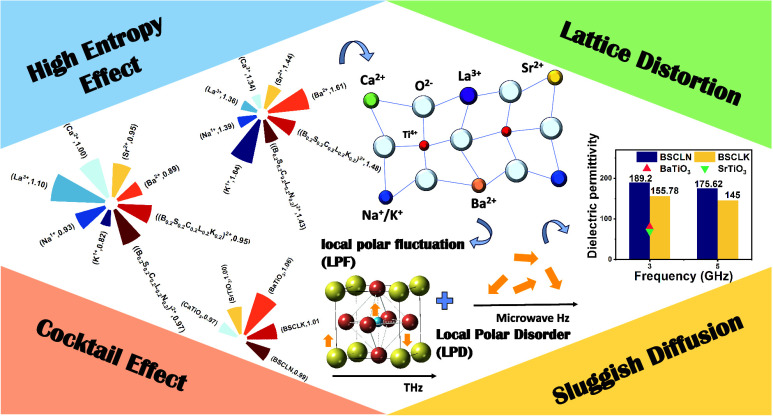

## 1 Introduction

High-entropy ceramics have become a promising class of materials, offering novel functionalities and their combinations for various applications such as thermoconductivity and piezoelectricity.^1–3^ High-entropy perovskite oxides are typically single-phase solid solutions containing five or more metal cations that occupy the same crystallographic site in near-equiatomic proportions. Their stability at high temperatures is driven by high configurational entropy, which lowers the Gibbs free energy and facilitates the formation of a single-phase structure.^4,5^ This characteristic provides opportunities to tailor dielectric properties through entropy-driven phase stabilization and lattice distortion effects.^6^ In principle, the interplay among chemical disorder, ionic polarizability and lattice vibrations governs the dielectric behavior of high-entropy perovskite oxides in the microwave and terahertz (THz) regimes, thereby influencing both dielectric dispersion and energy loss.^8,9^

Quantum paraelectrics are characterized by their proximity to a ferroelectric phase transition, where quantum fluctuations suppress the development of spontaneous polarization as the temperature approaches 0 K.^10,11^ Thus, these materials exhibit relatively high dielectric permittivity at low temperatures but do not undergo polarization saturation due to the persistence of quantum fluctuations. In classical quantum paraelectrics, such as SrTiO_3_ and KTaO_3_, the dielectric response is strongly influenced by soft phonon modes, specifically, the soft transverse optical (TO) phonon mode. This mode corresponds to a low-frequency polar lattice vibration whose frequency decreases as the system approaches a structural phase transition. The extent of this softening reflects the proximity of the system to a polar (ferroelectric) state.^12,13^ Such soft-mode behavior leads to pronounced temperature and frequency dependence of the dielectric properties. Owing to these unique characteristics, quantum paraelectrics have been widely investigated for applications including cryogenic capacitors, microwave filters, terahertz (THz) components, and capacitive sensors.^14–16^

Recently, high-entropy dielectric ceramics have attracted considerable attention due to their high dielectric permittivity and low dielectric loss. Their inherent advantages, including compositional flexibility, entropy-driven phase stabilization, and lattice distortion, have stimulated growing interest in their application in electronic devices such as piezoelectric and electrocaloric systems.^17–19^ However, despite the rapid development of high-entropy dielectric ceramics, their dielectric performance at high frequencies (GHz–THz regimes) remains insufficiently understood and is often limited by pronounced dielectric relaxation and increased energy dissipation. On the other hand, conventional quantum paraelectrics exhibit excellent dielectric stability but typically suffer from relatively low permittivity at room temperature due to suppressed polarization.^11^ These limitations highlight a fundamental challenge in dielectric materials design: achieving simultaneously high permittivity, low dielectric loss, and frequency stability across a broad frequency range. In particular, the interplay between structural disorder, polarization dynamics, and lattice vibrations in complex systems remains an open scientific question, especially in the GHz–THz regime.

Here, we address this challenge by introducing a new class of materials, namely high-entropy quantum paraelectrics (HEQPs), which combine entropy-induced structural disorder with quantum paraelectric fluctuations. In perovskite titanates (ATiO_3_), the multielemental doping of A-site cations with different valence states and ionic radii can generate local charge heterogeneity, lattice distortion, and local field fluctuations, thereby modifying polarization dynamics.^20,21^ It is worth noting that the relatively small and stable ionic radius of Ti^4+^ at the B site enables the formation of a robust TiO_6_ octahedral framework. This structural backbone can accommodate a wide range of A-site cations with different ionic radii and valence states, thereby allowing compositional flexibility without significantly compromising the overall structural integrity of the perovskite lattice.^22,23^

In this context, HEQP ceramics, (Ba_0.2_Sr_0.2_Ca_0.2_La_0.2_Na_0.2_)­TiO_3_ (BSCLN) and (Ba_0.2_Sr_0.2_Ca_0.2_La_0.2_K_0.2_)­TiO_3_ (BSCLK) were developed and their dielectric permittivity and loss were systematically investigated over a broad frequency range spanning kHz to THz. Both compositions exhibit a stable frequency response, maintaining relatively high dielectric permittivity and low loss across a wide frequency range. Notably, their dielectric performance in the GHz–THz regime is comparable to or exceeds that of conventional ferroelectric BaTiO_3_ and quantum paraelectric SrTiO_3_. By establishing this new class of materials that integrates high-entropy design with quantum paraelectric functionality, this work provides new insights into polarization dynamics in complex oxides and offers a promising strategy for the development of advanced dielectric materials for next-generation high-frequency applications.

## 2 Experimental Section

Ceramic samples of (Ba_0.2_Sr_0.2_Ca_0.2_La_0.2_Na_0.2_)­TiO_3_ (BSCLN) and (Ba_0.2_Sr_0.2_Ca_0.2_La_0.2_K_0.2_)­TiO_3_ (BSCLK) were prepared by the solid-state reaction method. BaCO_3_ (Alfa Aesar; purity = 99.8%), SrCO_3_ (Aldrich; purity ≥ 99.9%), CaCO_3_ (Alfa Aesar; purity = 99.5%), La_2_O_3_ (Sigma-Aldrich; purity ≥ 99.99%), TiO_2_ (Sigma-Aldrich; purity = 99.8%), K_2_CO_3_ (Alfa Aesar; purity ≥ 99.0%), Na_2_CO_3_ (Sigma-Aldrich; purity ≥ 99.5%) were used as starting materials. All raw powders were dried at 200 °C overnight to remove absorbed moisture before weighing according to the stoichiometric compositions. The powders were mixed by planetary ball milling (Pulverisette 5, Fritsch) at 180 rpm for 5 h using zirconia balls and ethanol as the milling medium. After drying, the mixed powders were calcined at 1100 °C for 4 h. The calcination temperature was selected based on preliminary trials at different temperatures, with XRD analysis confirming that this condition yields a single-phase perovskite structure. The calcined powders were ball-milled again for 5 h, and 5 wt % poly­(vinyl alcohol) (PVA) binder solution was added during the final 10 min of milling to improve powder compaction. The powders were pressed into pellets with a diameter of 13 mm and a thickness of 1 mm under 300 MPa. The pellets were then heated at 800 °C for 2 h to remove the binder. Sintering was carried out at 1350 °C for 2 h in closed alumina crucibles at a heating rate of 3 °C min^–1^, with the pellets covered by sacrificial zirconia powder.

The density of the sintered ceramics was measured by Archimedes method.^24^ For structural and phase analysis, the pellets were ground into fine powders. Crystal structure and phase composition were examined by X-ray diffraction (XRD, PANalytical X’Pert Pro diffractometer) over a 2θ range of 5–120° using Cu–K_α_ radiation. Raman spectroscopy was performed at room temperature using a LabRam microprobe system (ISA/Jobin-Yvon/Horiba, France) with a 633 nm Ar^+^ ion laser. The Raman spectra were deconvoluted using Gaussian peak functions with a Levenberg–Marquardt nonlinear least-squares fitting algorithm. The same fitting protocol, including baseline correction and peak profile, was consistently applied to all samples to ensure reliable comparison.

For electrical measurements, the pellets were polished to a thickness of 0.4 mm, and silver paste (Sun Chemical S.A. Ltd., C2050926P2) was applied to their major surfaces. Conductive electrodes were formed by firing the Ag paste at 450 °C for 30 min. The relative dielectric permittivity (ε′) and loss tangent (tan δ) were measured at room temperature over 100 Hz–1 MHz using a precision impedance analyzer (Agilent 4294A). Temperature-dependent dielectric permittivity and loss were measured using an LCR meter (Agilent 4284A). Polarization–electric field (*P*–*E*) loops and current–electric field (*I*–*E*) loops were recorded at 10 Hz using a ferroelectric tester (NPL, Teddington, UK) with a triangular waveform.

Microwave dielectric measurements were performed using a complementary split-ring resonator (CSRR) sensor equipped with an integrated heating circuit for temperature-controlled measurements (for details, see Figure S1 and the relevant information in Supporting Information).^25^ The permittivity and loss tangent were determined by fitting a CST Microwave Studio Suite 2023 model to the experimental data obtained from a vector network analyzer (VNA) (Figures S2–S4, Supporting Information).^25^ The control samples BaTiO_3_ and SrTiO_3_ were characterized using the same procedure to ensure consistent comparison. Terahertz dielectric properties were measured using a THz time-domain spectroscopy (THz-TDS) system.^26^ Measurements were performed in transmission mode to obtain the time-domain spectra. After Fourier transformation, the corresponding amplitude and phase spectra were obtained over 0.3–1 THz, from which the dielectric permittivity and loss were calculated.^27,28^

## 3 Results and Discussion

The Rietveld refinement of XRD data (Figure S5, Supporting Information) confirms that both compositions are single-phase materials with a cubic perovskite structure (Space Group, SG: *Pm*3̅*m*). The refined structural parameters (Table S1, Supporting Information) show that the unit cell volume of BSCLK is larger than that of BSCLN, which can be attributed to the larger ionic radius of K^+^ (1.64 Å), compared to Na^+^(1.39 Å). Based on the XRD results, both HEQP ceramics can be identified as centrosymmetric structures with space group *Pm*3̅*m*, with no evidence of a polar phase.

Ba^2+^, Sr^2+^ and Ca^2+^ are all alkaline earth elements known to enhance the dielectric properties of perovskite titanates.^18^ The aliovalent cation La^3+^ introduces local charge heterogeneity and contributes to lattice distortion, thereby enhancing local electric field fluctuations.^29^ Alkali cations Na^+^ and K^+^, with their relatively high polarizability were incorporated at the A-sites to promote short-range structural disorder.^30^ To evaluate the effect of multielement A-site substitution on the structural stability of the perovskite lattice, the Goldschmidt tolerance factor (*t*) was calculated for both compositions using the following expression:^22^

1 $$t=\frac{r_{A}+r_{O}}{\sqrt{2}\left(r_{B}+r_{O}\right)}$$

where *r*_A_, *r*_B_ and *r*_O_ are the ionic radii of the A-site cation, B-site cation and oxygen anion, respectively. For *t* ≈ 1, a stable perovskite structure is formed. Individually, La^3+^, Na^+^, and K^+^ are unable to form stable ATiO_3_ perovskite structures and are typically incorporated only as dopants at low concentrations.^30^ However, under high-entropy conditions, multiple A-site cations can be accommodated simultaneously. Using averaged ionic radii, the tolerance factors were calculated to be *t* = 0.99 for BSCLN and *t* = 1.014 for BSCLK. These values, being close to unity, indicate that the multicomponent A-site configuration can stabilize the perovskite structure. This suggests that, despite the high level of compositional complexity, the A-site cations can be effectively incorporated into the ATiO_3_ lattice. It should be noted that the use of averaged ionic radii in the tolerance factor calculation represents a first-order approximation for high-entropy systems. While this approach provides a useful estimate of the overall structural stability, it does not capture the local variations in ionic size and valence. Such local deviations can induce lattice distortion and contribute to the formation of local polar disorder/fluctuation in the present materials.

The Goldschmidt tolerance factors for SrTiO_3_, CaTiO_3_, and BaTiO_3_, together with the averaged values for BSCLN and BSCLK, indicate clear structural trends (Figure 1(a)). CaTiO_3_, with a tolerance factor significantly lower than unity, typically exhibits a distorted structure with octahedral tilting, whereas SrTiO_3_ (*t* ≈ 1) adopts a cubic structure and BaTiO_3_ (*t* > 1) shows ferroelectric lattice distortion. The high-entropy compositions have averaged tolerance factors close to unity, indicating a stabilized cubic-like structure despite compositional complexity. The selected A-site cations possess a wide range of electronegativities, from 0.93/0.82 (Na^+^/K^+^) to 1.10 (La^3+^) (Figure 1(b)). This variation introduces chemical heterogeneity within the A–O sublattice, leading to local variations in bonding characteristics. Such heterogeneity can perturb local electric fields and modify bond strengths,^7^ thereby broadening the distribution of local polar distortions. The A-site cations also exhibit a wide range of ionic radii from 1.34 Å (Ca^2+^) to 1.64 Å (K^+^) (Figure 1(c)). Their incorporation into the perovskite lattice introduces local strain fields, which further contribute to the spatial distribution of local polar distortions.^31^

**Figure 1:**
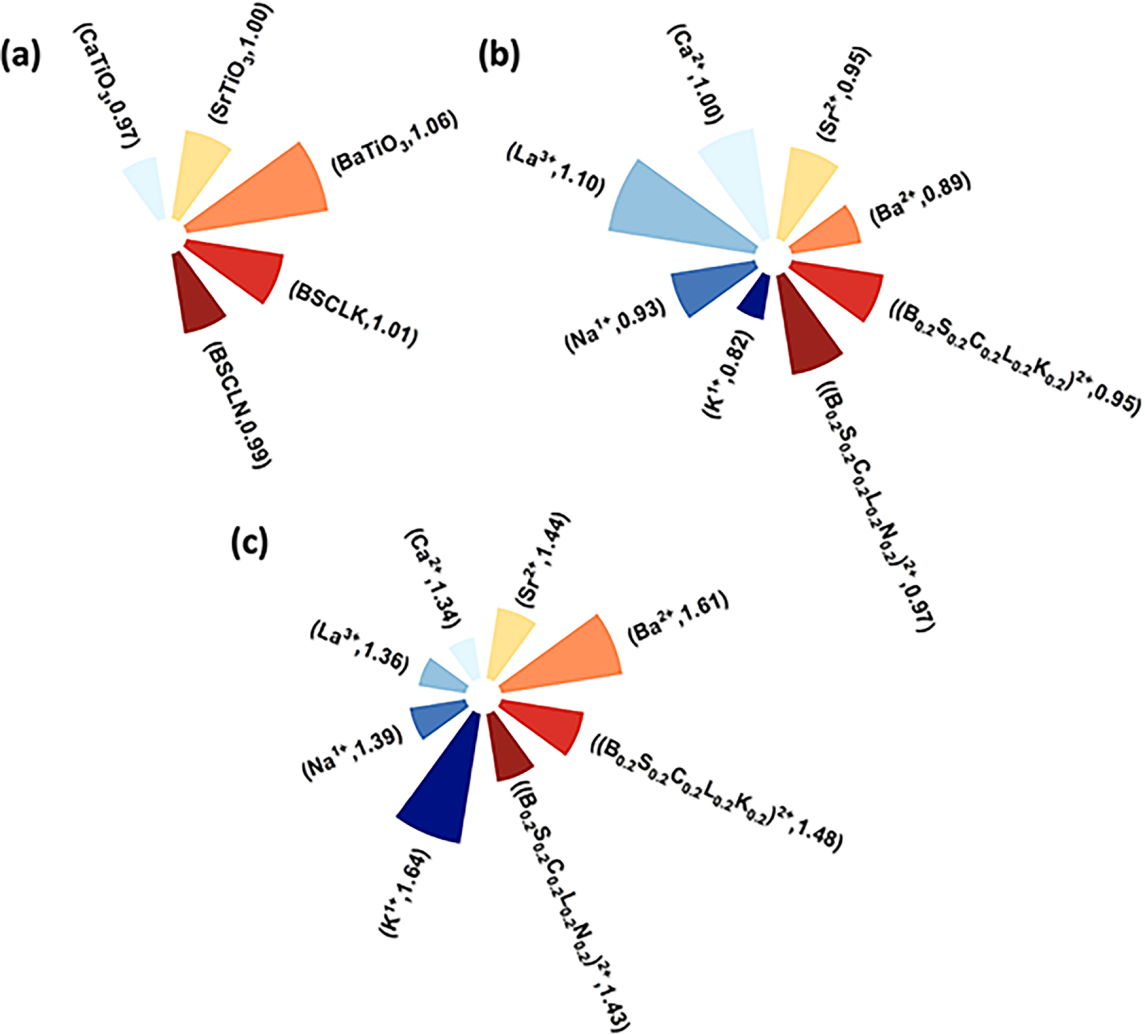
A graphic of (a) the Goldschmidt tolerance factors for SrTiO_3_, CaTiO_3_, BaTiO_3_, BSCLK and BSCLN, and (b) the electronegativity and (c) ionic radius of the A-site cations (Ba^2+^, Sr^2+^, Ca^2+^, La^3+^, Na^+^, K^+^), along with the calculated average values for BSCLN and BSCLK.^30^

The fitted Raman spectra of the BSCLN and BSCLK ceramics, together with the Raman-active phonon modes of the reference materials BaTiO_3_ and SrTiO_3_, reveal distinct vibrational characteristics (Figure 2). Because of the strong band broadening and overlap induced by compositional disorder in the high-entropy ceramics, the Raman peak fitting should be regarded as a supportive semiquantitative analysis. Nevertheless, the main fitted features are reproducible and remain consistent with the characteristic vibrational modes reported for related perovskite systems and with the comparative trends observed relative to BaTiO_3_ and SrTiO_3_.^32,33^ The HEQP ceramics exhibit distinct vibrational features arising from the collective influence of multicomponent A-site cations on lattice dynamics. In BaTiO_3_, the characteristic peak at ∼302 cm^–1^ is associated with tetragonal symmetry and long-range polar order.^34^ In SrTiO_3_, lattice-coupled local polar fluctuations (LPFs) are typically manifested by the transverse optical (TO) phonon mode at ∼240 cm^–1^.^35,36^ A broad Raman feature located at 265.4 cm^–1^ (BSCLN) and 246.0 cm^–1^ (BSCLK) (orange peaks in Figure 2) falls within a similar spectral region to the polar order-related mode reported for SrTiO_3_, suggesting comparable underlying lattice dynamics. These Raman shifts are slightly higher than those of the LPF mode in SrTiO_3_, showing a tendency toward the polar-state-related phonon mode of BaTiO_3_. This spectral evolution suggests an enhanced tendency toward ferroelectric ordering in the high-entropy quantum paraelectrics.^37^ Compared to BSCLN, BSCLK exhibits an additional feature at around 350 cm^–1^ (brown peak in Figure 2(b)). Given the larger average A-site ionic radius of BSCLK, the associated local structural distortion is expected to be stronger. Such distortion may lead to local symmetry lowering or symmetry breaking, which can activate Raman modes that are weak or inactive in a higher-symmetry perovskite lattice, as reported in compositionally complex perovskite systems.^18,38,39^ Therefore, the extra feature in BSCLK can be associated with enhanced local structural distortion and configurational local polar disorder.

**Figure 2:**
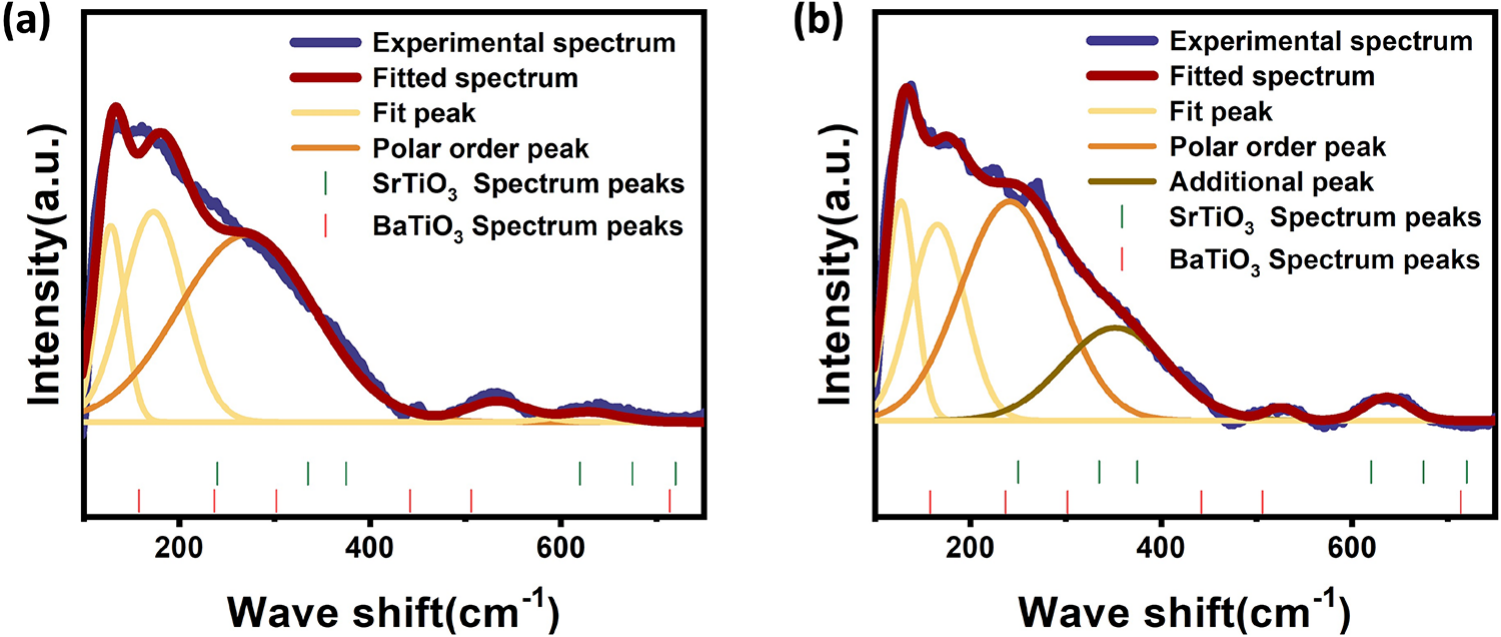
Fitted Raman spectra of (a) BSCLN, (b) BSCLK ceramics at room temperature; bottom panel: Raman-active modes of BaTiO_3_ and SrTiO_3_.^33,36^

The fitted Ba 3d, O 1s and La 3d XPS spectra of BaTiO_3_, BSCLN and BSCLK ceramics reveal clear differences in core-level features (Figures 3 and S6, Supporting Information). The Ba 3d core-level spectra of all three materials exhibit two main peaks corresponding to the 3d_5/2_ and 3d_3/2_ states. In BaTiO_3_, which contains a single A-site cation and a well-ordered perovskite lattice, these peaks are sharp and symmetric.^40^ In contrast, the Ba 3d spectra of the high-entropy BSCLN and BSCLK ceramics display noticeable peak broadening and slight shifts in binding energy. These spectral features indicate increased lattice distortion in the HEQP ceramics, arising from the random distribution of A-site cations with different ionic radii and valence states. The asymmetry of the broadened 3d_5/2_ and 3d_3/2_ peaks, together with the presence of satellite features, suggests multiple local chemical environments in which Ba atoms experience varying degrees of ionic and covalent bonding. Consistent with previous reports on high-entropy perovskite systems, such compositional complexity leads to local strain fields, symmetry breaking, and variations in bond lengths and bond angles within the BSCLN and BSCLK lattices.^20^ The O 1s and La 3d core level spectra further reflect the influence of compositional complexity (Figure S6, Supporting Information). The relative concentration of oxygen vacancies was evaluated by quantifying oxygen nonstoichiometry using a normalized oxygen spectral intensity approach. Specifically, the integrated area of the lattice oxygen peak, *A*(O_lattice_) was normalized to the area of the La3d_5/2_ peak, *A*(La3d_5/2_), which serves as a redox-invariant reference measured under identical conditions (inset, Figure S6). The normalized oxygen intensity ratio, *R*_O/Zr_, is defined as follows^41^

2 $$R_{O/\mathrm{Zr}}=\frac{A\left(O_{\mathrm{lattice}}\right)}{A\left({\mathrm{La}3d}_{5/2}\right)}$$

The calculated values of *R*_O/Zr_ for the BSCLN (∼3.70) and BSCLK (∼3.76) are comparable, indicating similar oxygen vacancy concentrations in both systems.

**Figure 3:**
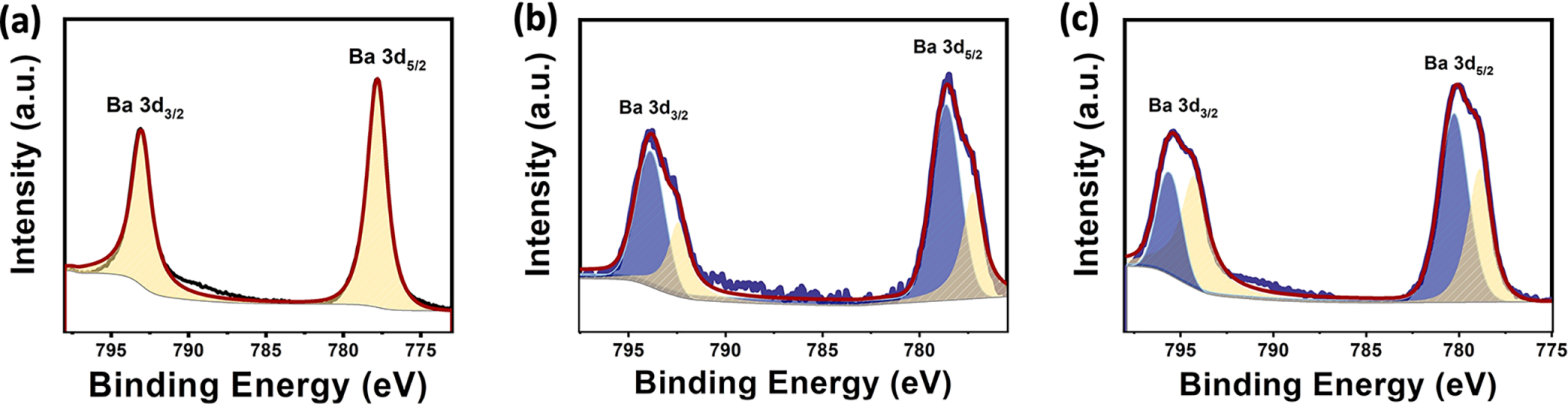
Fitted high-resolution Ba 3d core-level XPS spectra of (a) BaTiO_3_, (b) BSCLN and (c) BSCLK ceramics.

The temperature dependence of the relative dielectric permittivity and loss of the BSCLN and BSCLK ceramics, measured from 83 to 463 K at 500 Hz, 1 kHz, and 5 kHz, shows only weak frequency dispersion in this low-frequency regime (Figure 4(a,b)). These frequencies were therefore selected as representative points. It should be noted that all samples were synthesized under identical processing conditions, leading to comparable densification and microstructural features. Therefore, the influence of grain size on the dielectric properties is limited, and the observed dielectric behavior is mainly attributed to intrinsic polarization mechanisms associated with local polar disorder and lattice fluctuations. The gradual decrease in permittivity with increasing temperature indicates progressive disruption of local polar disorder and lattice-coupled fluctuations by thermal activation.^42,43^ Quantum fluctuations dominate at low temperatures, whereas thermal lattice vibrations become significant at higher temperatures; consequently, the system follows mean-field Curie–Weiss behavior only within this intermediate temperature range. In the intermediate temperature range 270–350 K, the dielectric permittivity of both BSCLN and BSCLK was therefore fitted using the Curie–Weiss law^11^

3 $$\varepsilon \left(T\right)=\frac{C}{T-T_{c}^{\mathrm{eff}}}\Rightarrow \frac{1}{\varepsilon \left(T\right)}=\frac{1}{C}T-\frac{T_{c}^{\mathrm{eff}}}{C}$$

where ε­(*T*) is the dielectric permittivity, *C* is the Curie constant, *T*_c_^eff^ is the extrapolated “virtual Curie temperature” corresponding to the hypothetical divergence point in the absence of quantum and disorder effects. The fitted values of *T*_c_^eff^ are −79 K for BSCLN and −86 K for BSCLK. The negative *T*_c_^eff^ values indicate the absence of a ferroelectric phase transition and are characteristic of a quantum paraelectric state in both compositions. Similar behavior has been widely reported in classical quantum paraelectrics such as SrTiO_3_.^44^ These results, together with the observed deviation from Curie–Weiss behavior at low temperatures, provide strong evidence for the quantum paraelectric nature of the present high-entropy ceramics. This further confirms that the incorporation of high-entropy disorder does not suppress, but rather coexists with, quantum paraelectric characteristics. Because the potential phase transition in BSCLN is closer to room temperature, the transverse optical (TO) phonon mode associated with local polar fluctuations exhibits a higher Raman shift (∼265.4 cm^–1^, Figure 2(a)) compared to BSCLK (∼246.0 cm^–1^, Figure 2(b)), indicating more active lattice-coupled LPFs at room temperature.^45^

**Figure 4:**
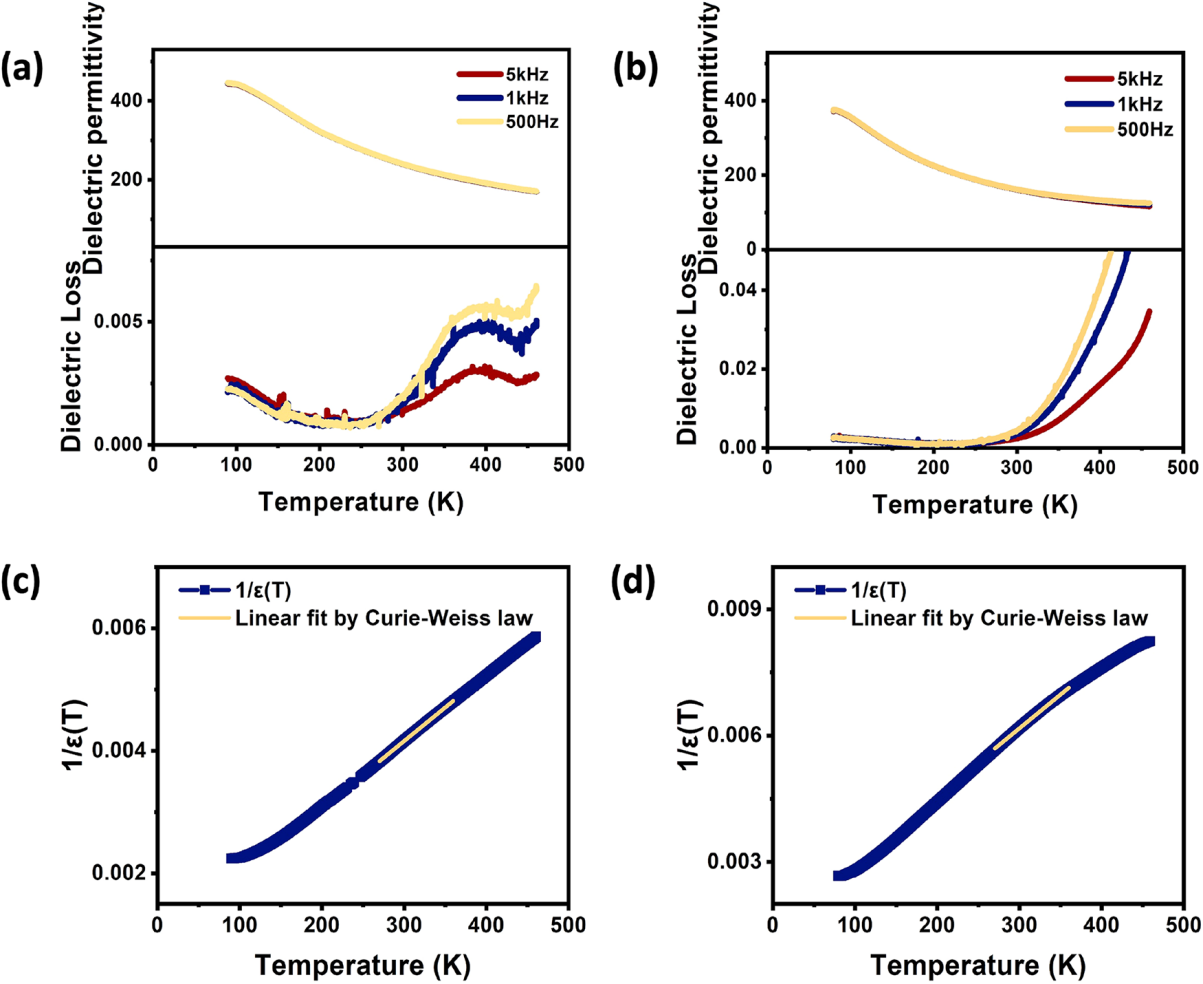
Temperature dependence of the relative dielectric permittivity and loss of (a) BSCLN and (b) BSCLK ceramics measured from 83 to 463 K at 500 Hz, 1 kHz and 5 kHz; (c, d) inverse permittivity as a function of temperature for BSCLN and BSCLK, respectively, measured at 1 kHz, with Curie–Weiss fits shown as solid yellow lines.

The dielectric loss of the BSCLN and BSCLK ceramics exhibits a nonmonotonic temperature dependence (Figure 4(a,b)). In BSCLN, a noticeable feature appears in the intermediate temperature range (∼300–400 K), which can be attributed to thermally activated relaxation processes associated with local polar disorder (LPD) and its interaction with lattice-coupled local polar fluctuations (LPFs). In contrast, the corresponding feature in BSCLK is less pronounced (Figure 4(b)), which can be attributed to a broader distribution of relaxation times arising from enhanced compositional disorder. In addition, the overall dielectric loss values in BSCLK are higher, which leads to a more gradual variation with temperature and masks the formation of a distinct peak. Despite these differences, both compositions exhibit a similar overall trend, characterized by an initial decrease in dielectric loss at low temperatures followed by a pronounced increase at higher temperatures. The reduction in loss at low temperatures is associated with the suppression of quantum fluctuations, which enables a more dynamic response of lattice-coupled local polar fluctuations to the applied field.^46^ At higher temperatures, the increase in dielectric loss is governed by thermally activated processes, particularly enhanced charge carrier mobility, leading to increased energy dissipation.^47,48^

The polarization–electric field (*P*–*E*) loops and corresponding current–electric field (*I*–*E*) curves of the BSCLN and BSCLK ceramics were measured at 10 Hz (Figure 5(a–d)). This frequency provides sufficient time for polarization switching and minimizes leakage contributions associated with prolonged voltage application at lower frequencies.^49^ The *P*–*E* loops are slim and nearly linear over the applied electric field range of ±5 MV m^–1^, with almost zero remanent polarization (*P*_r_) and coercive field (*E*_c_). The relative dielectric permittivity at the maximum applied electric field (*E*_max_) was calculated from the *P*–*E* loops using^50^

4 $$\varepsilon_{r}=\frac{dD_{\max}}{\varepsilon_{0}dE_{\max}}$$

where *D*_max_ is the maximum dielectric displacement of free charges and ε_0_ is the vacuum permittivity (8.854 × 10^–12^ F m^–1^). The calculated ε_r_ values for BSCLN and BSCLK are 247.4 and 261.7, respectively. In the HEQP ceramics, both high-entropy-enhanced LPD and LPFs are expected to contribute to the dielectric permittivity. Raman analysis indicates stronger disorder in BSCLK, likely arising from its larger average A-site ionic radius. Compared with LPFs, LPD has a much longer relaxation time and therefore contributes more strongly to the dielectric response at lower frequencies, leading to the higher permittivity of BSCLK. The corresponding *I*–*E* loops (Figure 5(c,d)) are symmetric, with no evidence of current peaks associated with domain switching or leakage-current-dominated conduction.^51,52^ This indicates that, although LPFs remain dynamic, the LPD does not condense into long-range ferroelectric order, which would otherwise be reflected by current peaks in the *I*–*E* curves.^46,53^

**Figure 5:**
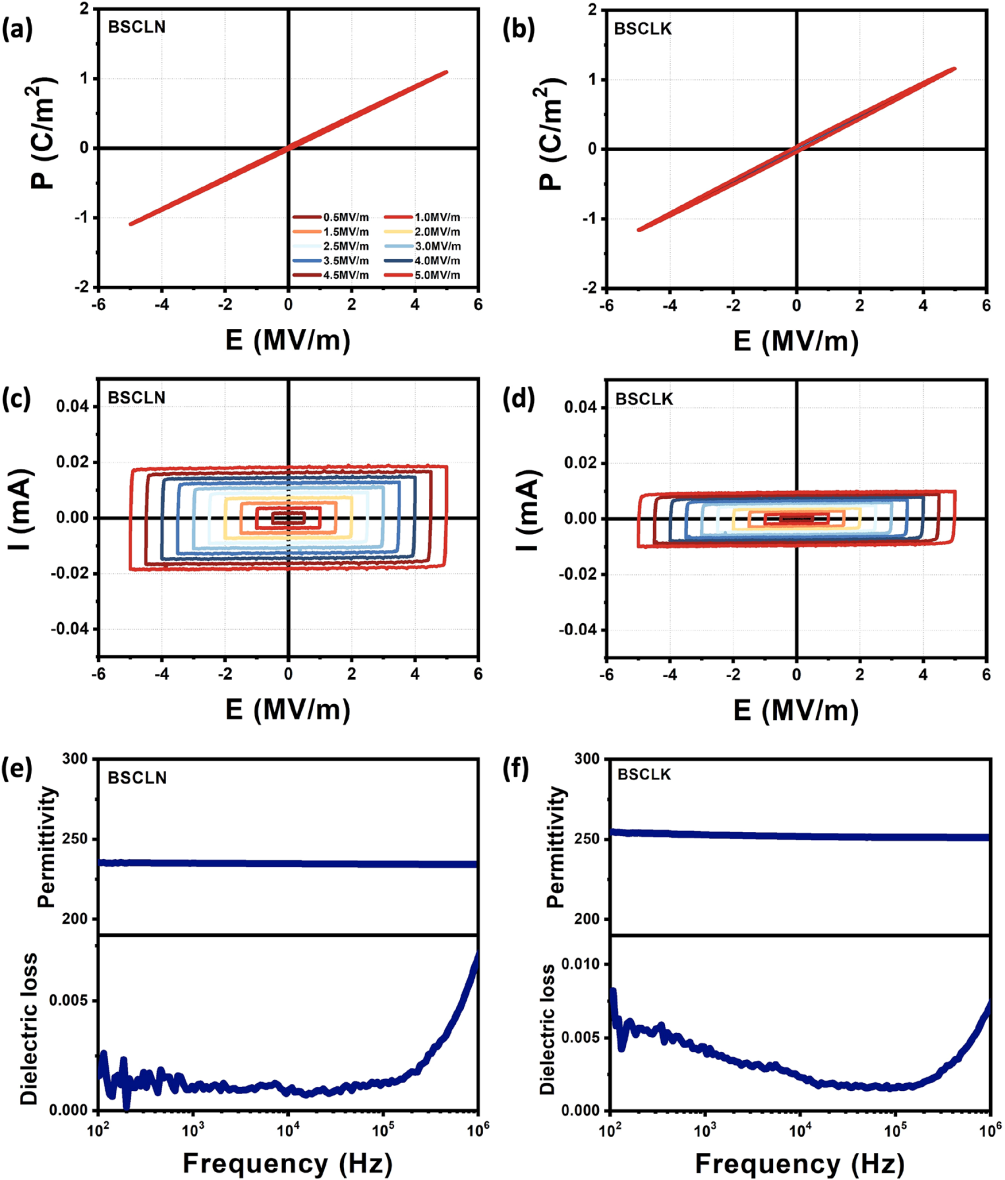
(a, b) *P*–*E* loops of the BSCLN and BSCLK ceramics; (c, d) *I*–*E* loops of the BSCLN and BSCLK ceramics; (e, f) the frequency dependencies of the relative dielectric permittivity and loss of the BSCLN and BSCLK ceramics, respectively.

The dielectric permittivity and loss of BSCLN and BSCLK exhibit weak frequency dependence in the low radio frequency (RF) range (Figure 5(e,f)). The dielectric permittivity remains nearly constant at ∼235 for BSCLN and ∼255 for BSCLK over the frequency region from 100 Hz to 1 MHz. This weak dispersion indicates that the polarization response is dominated by local polar disorder (LPD) rather than the dynamics of long-range ferroelectric domains.^54^ LPD consists of spatially confined and weakly correlated polar regions that can reorient relatively freely under an alternating electric field.^42^ The results are in good agreement with the ferroelectric hysteresis results (*P*–*E* loops) reflecting a field-independent permittivity. The absence of domain-switching features in the *I*–*E* curves further supports this interpretation. Moreover, the discontinuous nature of LPD enables polarization reorientation with minimal energy dissipation, resulting in very low dielectric loss (<0.005).^42^ Although XPS analysis indicates the presence of oxygen vacancies in both BSCLN and BSCLK, these defects are largely immobile at room temperature and therefore do not contribute significantly to dielectric loss.^55,56^ A slightly higher loss in BSCLK can be attributed to enhanced LPD relaxation associated with its increased structural disorder.

The dielectric permittivity and loss of BSCLN and BSCLK show clear frequency-dependent behavior at microwave frequencies and room temperature (Figure 6). The resonance responses of both compositions differ between the 3 and 5 GHz ranges, particularly in the depth and sharpness of the resonance features (Figure 6(a–d)). In general, the response at 3 GHz is more pronounced, whereas the feature at 5 GHz becomes broader and more gradual. This behavior suggests a decrease in the mechanical quality factor (*Q*) with increasing frequency, which may contribute to enhanced energy dissipation at higher GHz frequencies.^57^ Under microwave irradiation, the polarization response of HEQPs is mainly governed by entropy-enhanced LPFs with short relaxation times. However, LPD, which has a longer relaxation time, remains partially active in the GHz regime and contributes to additional energy dissipation through relaxation loss.

**Figure 6:**
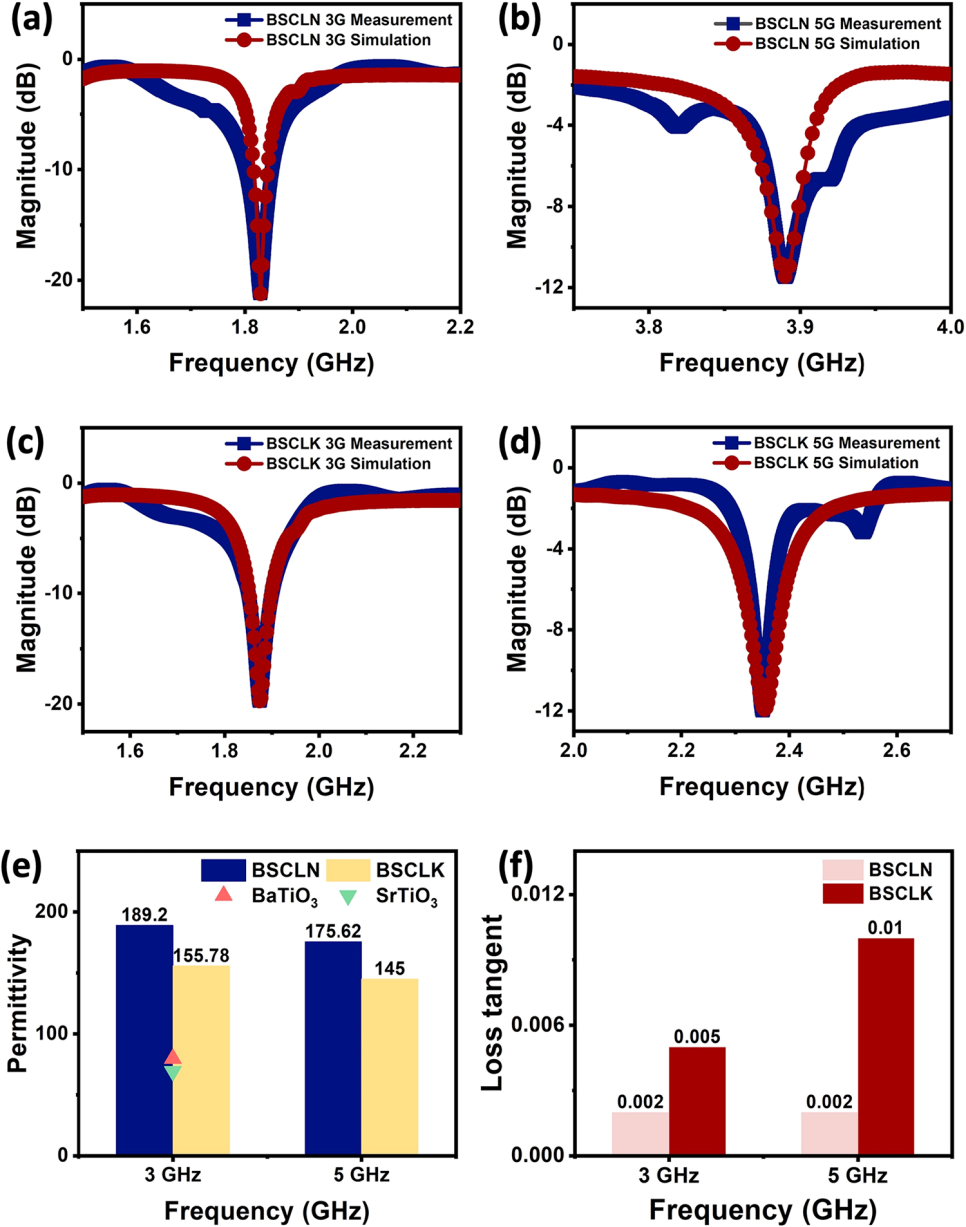
Microwave testing and simulations of (a, b) BSCLN and (c, d) BSCLK ceramics at 3 and 5 GHz; (e) a comparison of the calculated dielectric permittivity at 3 and 5 GHz for BSCLN, BSCLK and reference materials of BaTiO_3_ and SrTiO_3_; (f) loss tangent of the BSCLN and BSCLK ceramics at 3 and 5 GHz.

At 3 GHz, both BSCLN and BSCLK exhibit dielectric permittivity values (∼155–189) more than twice those of the reference BaTiO_3_ (∼79.4) and SrTiO_3_ (∼69.46) samples measured under identical conditions (Figure 6(e)). This comparison confirms the enhanced microwave dielectric response of the HEQP ceramics. In BaTiO_3_, long-range ferroelectric order is relatively rigid at microwave frequencies, limiting its polarization response. In contrast, both LPD and entropy-enhanced LPFs remain active in BSCLN and BSCLK, contributing to their higher permittivity.^58^ Compared with SrTiO_3_, the HEQP ceramics possess more dynamic entropy-enhanced LPFs, further increasing their dielectric response. It is important to note that the improved microwave dielectric performance of the HEQPs cannot be solely attributed to compositional modification of BaTiO_3_- or SrTiO_3_-based systems. Instead, the high-entropy design introduces a fundamentally different polarization landscape, where entropy-induced local polar disorder (LPD) and lattice-coupled local polar fluctuations (LPF) coexist and interact. This synergistic mechanism enables dynamic polarization responses over a broad frequency range, in contrast to the rigid domain response in ferroelectrics or the weak polarization in conventional quantum paraelectrics. As a result, the HEQPs achieve a unique combination of relatively high permittivity and low dielectric loss at GHz frequencies, demonstrating a distinct advantage over traditional dielectric materials.

A gradual decrease in permittivity with increasing frequency from 3 to 5 GHz is observed for both ceramics. As the frequency of the applied electric field exceeds the characteristic relaxation frequency of the LPD, these polar entities can no longer follow the rapidly oscillating field, leading to a reduced contribution to the dielectric permittivity.^59^ At the same time, the lag between the polarization response and the applied field leads to increased energy dissipation, giving rise to enhanced dielectric loss.^59^ This behavior is consistent with the trend observed in the RF band (Figure 5(e–f)), where dielectric loss increases with frequency due to the delayed response of polarization dynamics. BSCLN exhibits higher permittivity than BSCLK in both the 3 and 5 GHz ranges (Figure 6(e)). Since *T*_c_^eff^ of BSCLN is closer to room temperature than that of BSCLK, the LPFs in BSCLN are more active and therefore contribute more strongly to the dielectric response.^58^ In contrast, BSCLK shows higher dielectric loss at GHz frequencies (Figure 6(f)), which can be attributed to enhanced LPD relaxation associated with the larger K^+^ ionic radius and stronger local structural disorder.

The temperature-dependent microwave dielectric properties of BSCLN and BSCLK were further investigated in the 3 GHz region (Figure 7), with the corresponding resonance characteristics provided in the Supporting Information (Figure S7­(a–l)). Both compositions exhibit clear temperature-dependent dielectric behavior. The dielectric permittivity decreases continuously as the temperature increases from 298 to 423 K. Upon heating, thermal fluctuations increasingly disturb LPFs and LPD, weakening their contribution to polarization in the GHz band and thereby reducing the microwave permittivity.^37,46^

**Figure 7:**
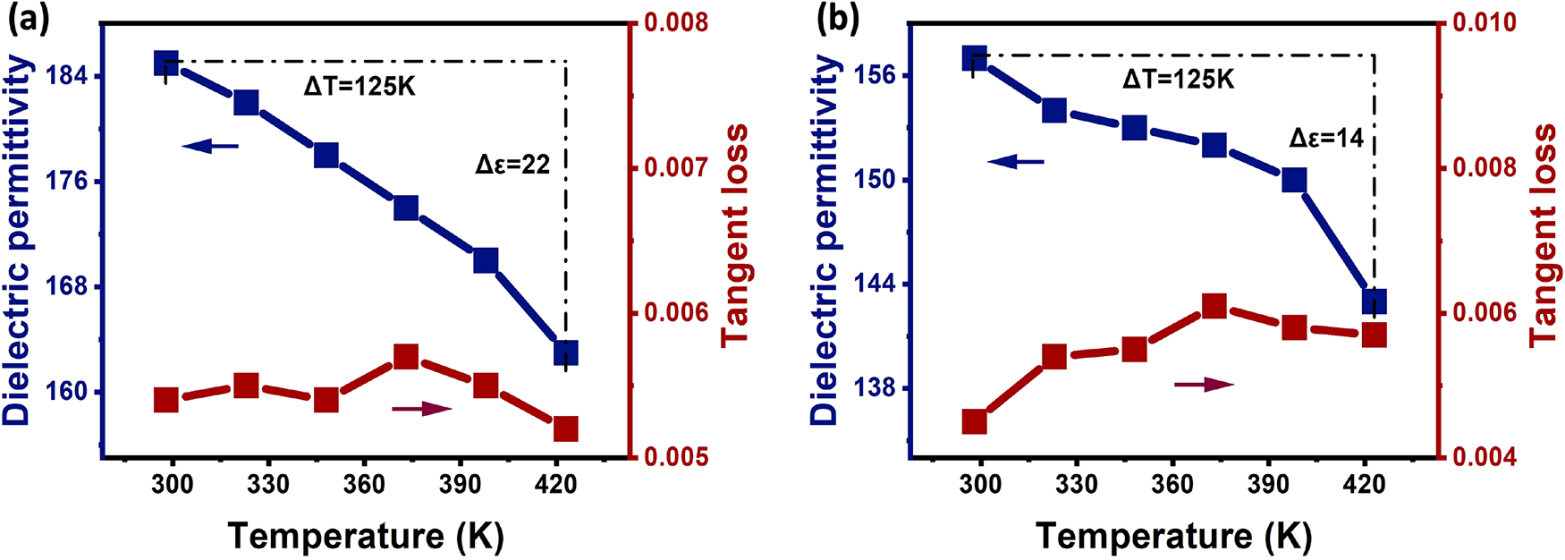
Temperature dependence of dielectric permittivity and loss tangent of (a) BSCLN and (b) BSCLK ceramics measured at 3 GHz.

Within the same temperature range, BSCLN exhibits a larger variation in dielectric permittivity than BSCLK, suggesting that its highly active LPFs are more sensitive to temperature changes. In quantum paraelectrics such as SrTiO_3_, the lattice-coupled LPF or soft-mode response has been reported to harden noticeably with increasing temperature, leading to pronounced changes in dielectric permittivity.^11^ In contrast, BSCLK shows a less temperature-sensitive dielectric response, which can be associated with a harder LPF mode and a more negative *T*_c_^eff^, resulting in improved dielectric stability over the same temperature range.^37,60^ The dielectric loss of BSCLN remains nearly constant upon heating, whereas BSCLK exhibits a slight increase in loss. This increase can be attributed to enhanced LPD relaxation at elevated temperatures, which increases energy dissipation under microwave excitation.

The dielectric behavior of BSCLN and BSCLK was further investigated in the terahertz (THz) range of 0.3–1.0 THz (Figure 8). In this regime, the dielectric response of the HEQPs is primarily governed by LPFs. The more active LPFs in BSCLN, associated with its higher *T*_c_^eff^ (closer to room temperature, compared with BSCLK), lead to higher permittivity than that of BSCLK. BSCLN maintains a relatively stable permittivity across the measured THz range, indicating more coherent lattice dynamics dominated by a well-defined LPF mode. In contrast, BSCLK shows a gradual increase in permittivity with frequency, suggesting stronger frequency dispersion associated with a broader distribution of relaxation processes.^59,61,62^

**Figure 8:**
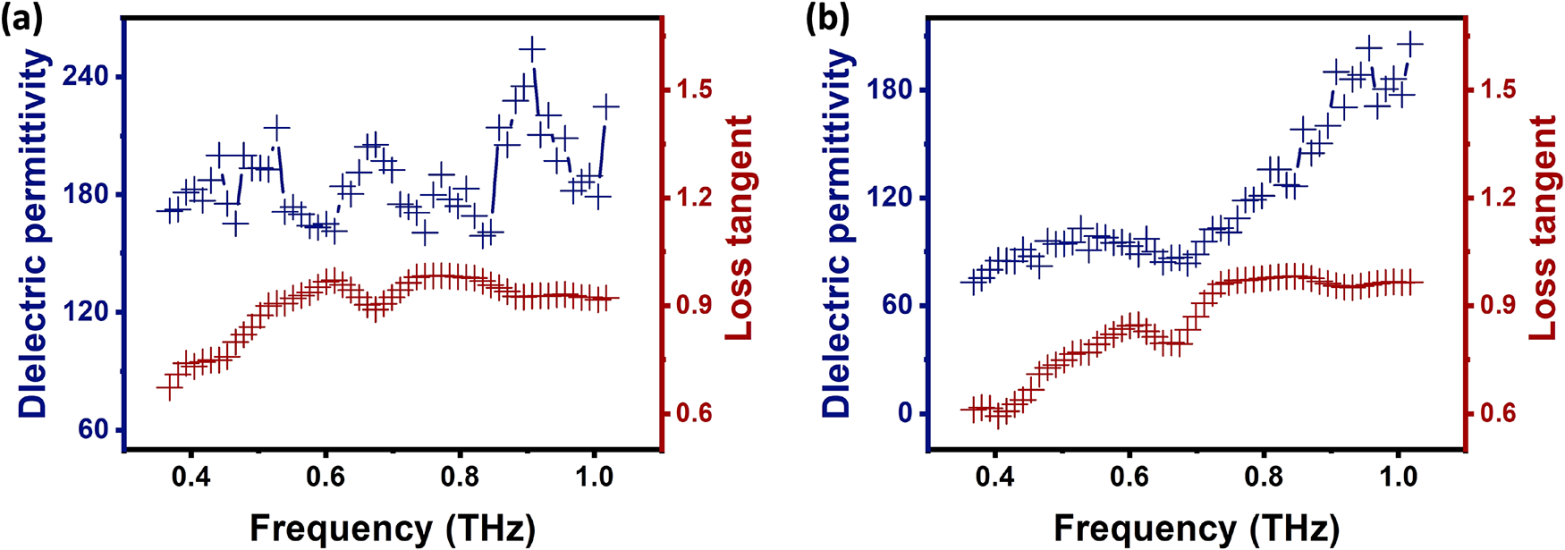
Dielectric permittivity and loss tangent of (a) BSCLN and (b) BSCLK ceramics measured by THz spectroscopy over 0.3–1.0 THz.

The dielectric loss increases markedly from the microwave to the THz regime, particularly in BSCLN, where the loss rises from ∼0.002 in the microwave range to ∼0.6 in the THz range. At GHz frequencies, the applied field frequency is far below that of the LPF mode (∼1–3 THz), resulting in limited phonon-related energy dissipation. However, in the THz regime, the dielectric response is dominated by the interaction between the applied field and lattice-coupled local polar fluctuations (LPFs) modes. As the frequency of the applied field approaches that of the LPF mode, the dielectric loss increases significantly due to enhanced absorption associated with the imaginary part of the dielectric function (ε″). This behavior is characteristic of phonon-related dielectric response, where the loss peak arises near the transverse optical (TO) phonon frequency as a result of damping of the lattice vibrations.^43,63,64^ Consequently, both compositions exhibit an increasing dielectric loss with frequency in the THz range, reflecting the proximity between the excitation frequency and the intrinsic polar lattice dynamics.

## 4 Conclusions

In this work, novel high-entropy quantum paraelectric (HEQP) ceramics, (Ba_0.2_Sr_0.2_Ca_0.2_La_0.2_Na_0.2_)­TiO_3_ (BSCLN) and (Ba_0.2_Sr_0.2_Ca_0.2_La_0.2_K_0.2_)­TiO_3_ (BSCLK), were successfully synthesized and their dielectric properties were systematically investigated from the RF to THz regimes. Both compositions exhibit a cubic perovskite structure (*Pm*3̅*m*), negative extrapolated Curie temperatures (<0 K), and slim *P*–*E* loops, confirming their intrinsic quantum paraelectric nature. The high-entropy design introduces configurational local polar disorder (LPD) and enhances lattice-coupled local polar fluctuations (LPFs), resulting in a stable polarization response. The HEQP ceramics exhibit relatively high dielectric permittivity (∼200) and ultralow loss (<0.002) in the low-frequency (kHz–MHz) regime, while maintaining good dielectric stability into the GHz–THz range. In the microwave regime, the dielectric response is primarily governed by entropy-enhanced LPFs, whereas in the THz regime, it is dominated by phonon-related dynamics associated with LPFs, accompanied by increased dielectric loss. Between the two compositions, BSCLN exhibits superior dielectric performance in the GHz–THz range, which is attributed to more active LPFs, consistent with the Raman analysis.

By integrating high-entropy structural design with quantum paraelectric functionality, this work provides new insight into the interplay between configurational disorder and polarization dynamics and establishes a promising strategy for the development of advanced dielectric materials for high-frequency microwave and terahertz applications.

## Supplementary Material



## Notes

Experimental details of the temperature-controlled CSRR measurement system and CST simulation procedure; calibration of unloaded CSRR response; temperature-dependent CSRR measurements; Rietveld refinement of XRD patterns; refined structural parameters; high-resolution XPS spectra; temperature-dependent microwave resonance behavior at 3 GHz (PDF)
