# Supplementary material for: High-Entropy Design of Perovskite Quantum Paraelectrics with Improved Dielectric Properties in GHz and THz Bands
Source: ACS Appl Mater Interfaces. 2026 Jun 22;18(25):35575–86. doi: 10.1021/acsami.6c04586 (PMC13339006; doi:10.1021/acsami.6c04586)
Supplement: Supplementary file 1 [file am6c04586_si_001.pdf]

SUPPORTING INFORMATION

High-entropy design of perovskite quantum paraelectrics with improved dielectric properties in GHz and THz bands

Wanting Hu[a], Xuyao Tang[a], Harry Baxter[b], Vladimir Koval[c], Krishnarjun Banerjee[a], Michael J. Reece[a], Bin Yang[b], Haixue Yan*[a]*

[a] School of Engineering and Materials Science, Queen Mary University of London, Mile End Road, London E1 4NS, UK

[b] Faculty of Science and Engineering, University of Chester, Chester CH2 4NU, UK

[c] Institute of Materials Research, Slovak Academy of Sciences, Watsonova 47, 040 01 Kosice, Slovakia

Corresponding author.

** Haixue Yan - E-mail: h.x.yan@qmul.ac.uk*

**Bin Yang - E-mail: b.yang@chester.ac.uk*

Complementary Split Ring Resonators (CSRRs) are intricate structures with a distinctive pattern that exhibits resonant behaviour primarily in the microwave frequency range. These resonators consist of two interlocking rings - one featuring a gap and the other with a bridge which disrupts the normal transmission of a microstrip line to achieve a specific resonance frequency. The temperature-controlled CSRR sensor incorporates two heating elements attached to the sample test area, each regulated by dedicated power supplies. By adjusting the voltage and current across the heating elements, precise temperature states can be maintained within the CSRR system. The system was connected to a vector network analyser (VNA) to collect transmission data (Figure S1), while the samples were modelled in a physics engine simulation software package CST. Initially, arbitrary values of dielectric permittivity and loss tangent were assigned in the model before optimisation. Through iterative parameter sweeps of the simulated permittivity and loss tangent, a correlation was established between the changes in permittivity/loss and the shifts in the resonance frequencies and attenuations (Figure S2). Following the inverse square law, this nonlinear relationship was linearised into the form of $y = mx + c$. By inputting the measured resonance frequency data into this equation, the calculated dielectric permittivity can be achieved. Similarly, the loss tangent was derived by applying the measured resonance Q factor to a corresponding linear function (with distinct m and c values).

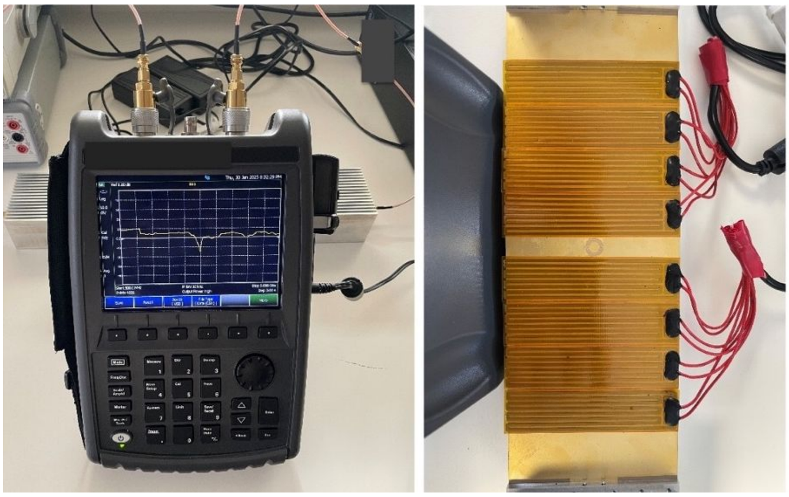

Figure S1. VNA (left) and heated CSRR with unloaded sample.

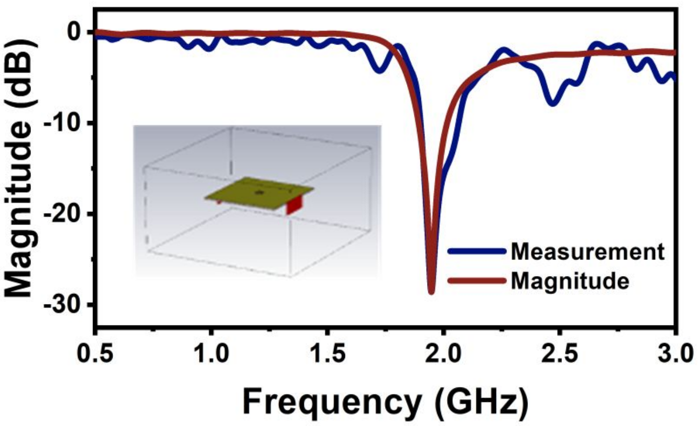

Figure S2. Verification of unloaded measurement data from VNA with the simulated results from CST (inset plot).

The temperature-controlled CSRR was first tested unloaded while heating to 298, 323, 348, 373, 398, and 423 K. As shown in Figure S3, the dielectric behaviour of CSRR was only minimally affected by the increase in temperature. Nevertheless, even these slight deviations were calibrated into the CST model to ensure an accurate initial fit for the unloaded system.

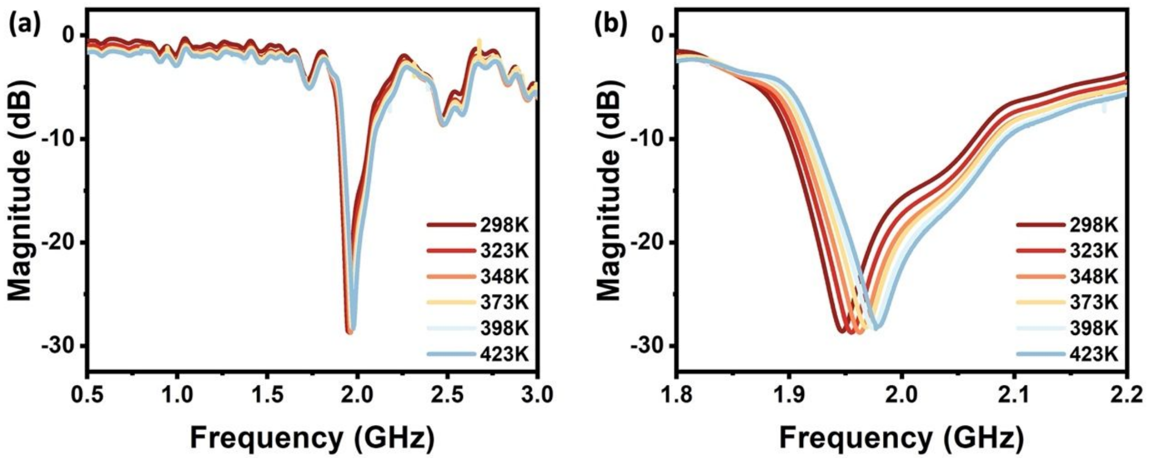

Figure S3. Effect of temperature change on the unloaded CSRR sensor (a) Full frequency range from 0.5 to 3.0 GHz. (b) Enlarged view of the resonance dip around 2.0 GHz.

The heating circuit is switched on, and the sample temperature is monitored using an infrared laser thermometer, as shown in Figure S4. The iterative method to characterise permittivity and loss tangent is exactly the same as the room temperature CSRR.

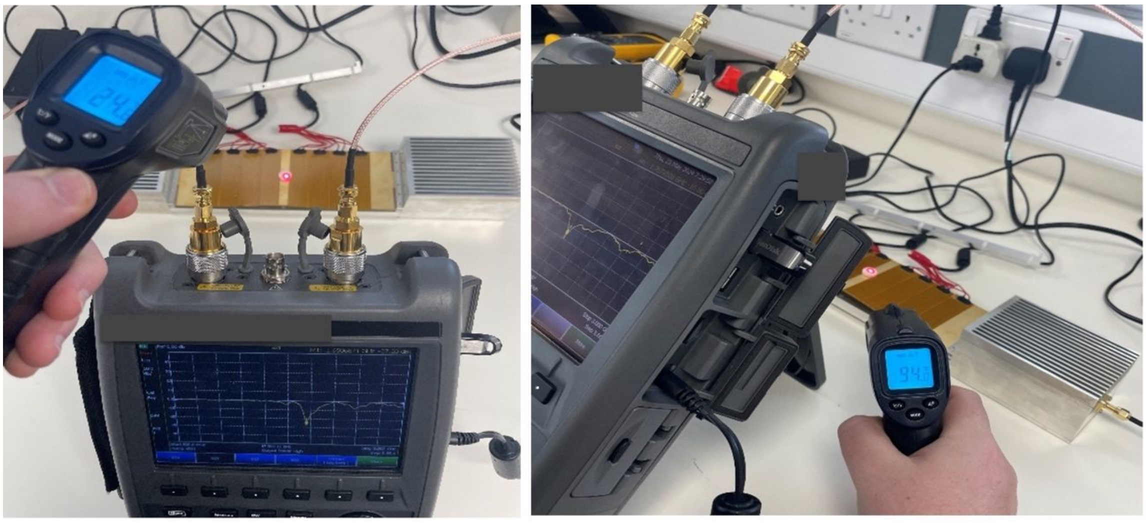

Figure S4. CSRR sample test under various temperatures.

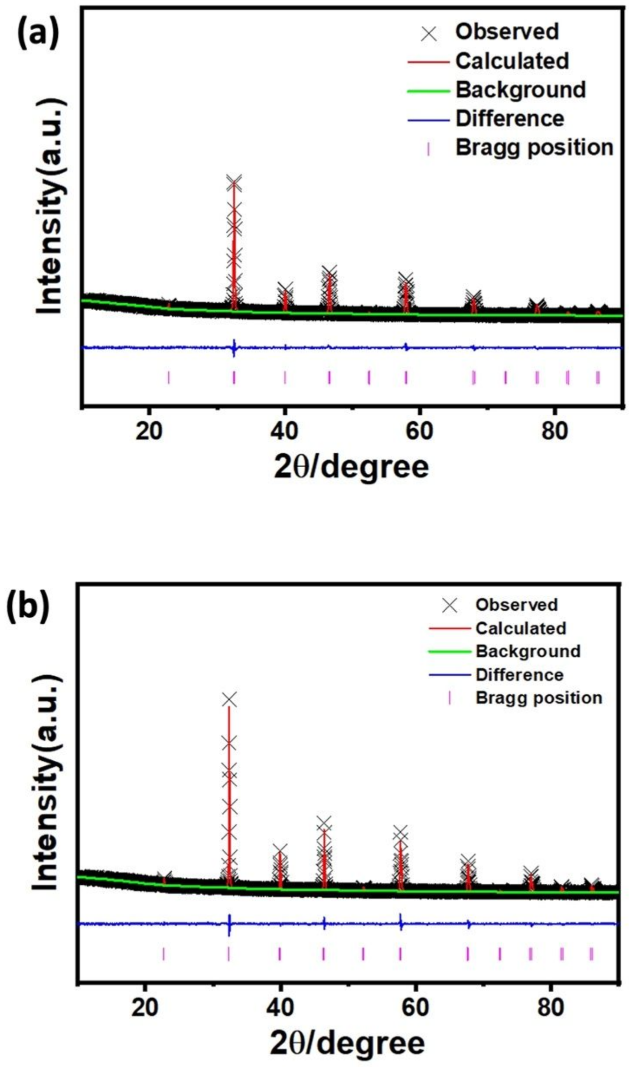

Figure S5. The Rietveld-refined XRD patterns of (a) BSCLN and (b) BSCLK ceramics.

Table S1. The refined structure parameters and reliability R_{wp} factor for the BSCLN and BSCLK ceramics.

**Composition**	**Space group**	a [Å]	b [Å]	c [Å]	Unit volume [Å^3]	$\boldsymbol{R_{wp}}$
BSCLN	$Pm\bar{3}m$	3.9058	3.9058	3.9058	59.6	4.38
BSCLK	$Pm\bar{3}m$	3.9162	3.9162	3.9162	60.1	4.68

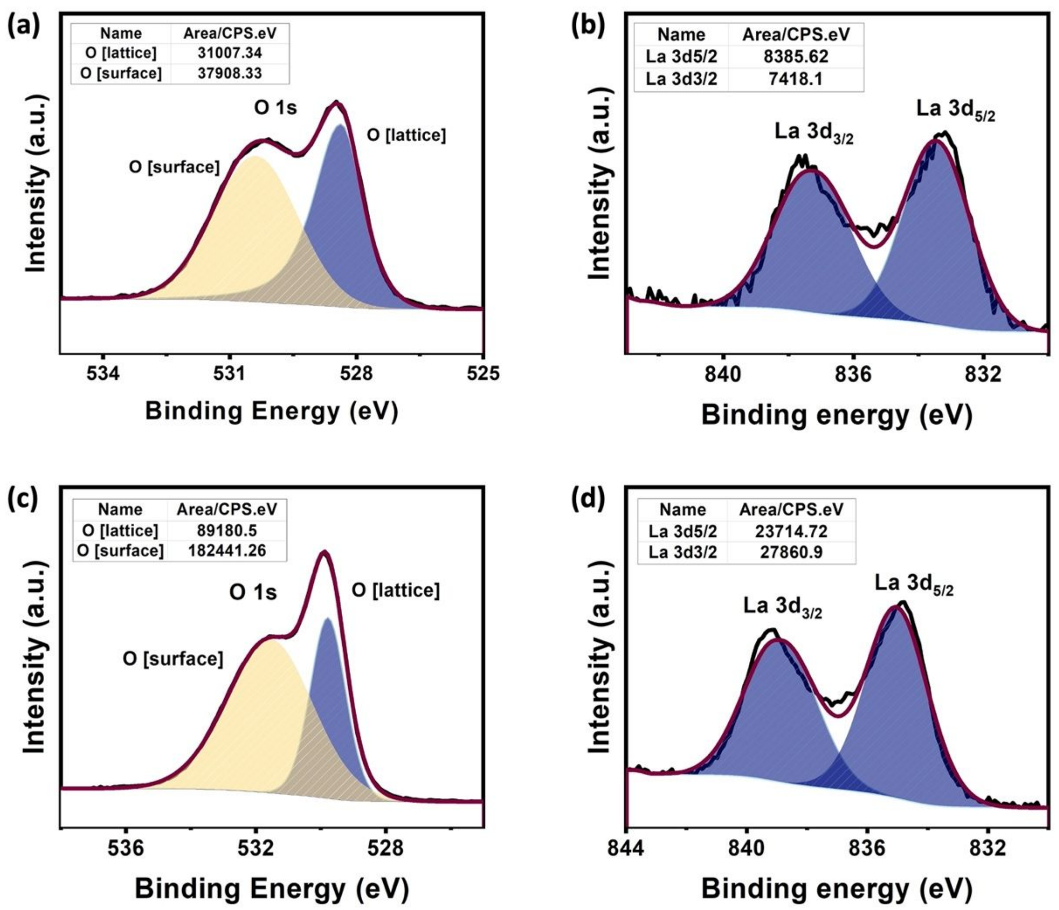

Figure S6. The fitted high-resolution XPS spectra of core levels of (a) O 1s and (b) La 3d states for the BSCLN ceramic, and (c) O 1s and (d) La 3d states for the BSCLK sample.

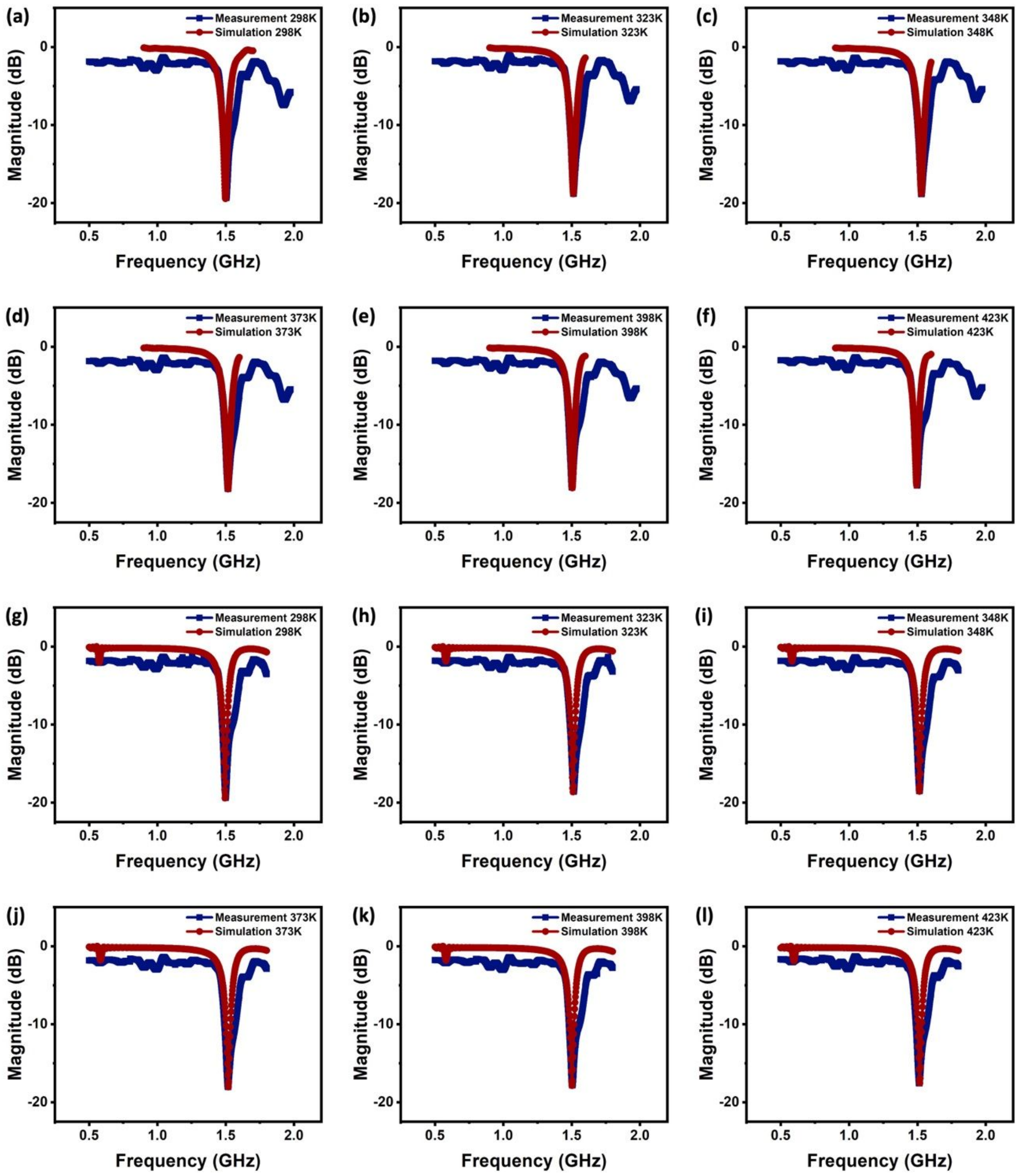

Figure S7. The resonance plots of (a–f) BSCLN and (g–l) BSCLK ceramics at 3 GHz, as collected at different temperatures in the range 298–423 K.
